# Rainfall during parental care reduces reproductive and survival components of fitness in a passerine bird

**DOI:** 10.1002/ece3.1345

**Published:** 2014-12-24

**Authors:** Meit Öberg, Debora Arlt, Tomas Pärt, Ane T Laugen, Sönke Eggers, Matthew Low

**Affiliations:** 1 Department of Ecology, Swedish University of Agricultural SciencesBox 7044, Uppsala, 75007, Sweden; 2 Aronia Coastal Zone Research Team, Novia University of Applied Sciences & Åbo Academy UniversityRaseborgsvägen 9, Ekenäs, 10600, Finland

**Keywords:** Feeding frequency, offspring, precipitation, provisioning, weather, wheatear

## Abstract

Adverse weather conditions during parental care may have direct consequences for offspring production, but longer-term effects on juvenile and parental survival are less well known. We used long-term data on reproductive output, recruitment, and parental survival in northern wheatears (*Oenanthe oenanthe*) to investigate the effects of rainfall during parental care on fledging success, recruitment success (juvenile survival), and parental survival, and how these effects related to nestling age, breeding time, habitat quality, and parental nest visitation rates. While accounting for effects of temperature, fledging success was negatively related to rainfall (days > 10 mm) in the second half of the nestling period, with the magnitude of this effect being greater for breeding attempts early in the season. Recruitment success was, however, more sensitive to the number of rain days in the first half of the nestling period. Rainfall effects on parental survival differed between the sexes; males were more sensitive to rain during the nestling period than females. We demonstrate a probable mechanism driving the rainfall effects on reproductive output: Parental nest visitation rates decline with increasing amounts of daily rainfall, with this effect becoming stronger after consecutive rain days. Our study shows that rain during the nestling stage not only relates to fledging success but also has longer-term effects on recruitment and subsequent parental survival. Thus, if we want to understand or predict population responses to future climate change, we need to consider the potential impacts of changing rainfall patterns in addition to temperature, and how these will affect target species' vital rates.

## Introduction

The recent focus on the response of organisms' phenologies to increasing spring temperatures (Crick and Sparks 1999; Sparks 1999; Parmesan and Yohe 2003; Lehikoinen et al. 2004; Visser et al. 2004; Chambers et al. 2013) has largely been because of expected temperature-associated changes in reproductive output and population growth rates (exemplified by studies on birds: Visser et al. 1998; Both et al. 2006; Møller et al. 2008; but see Reed et al. 2013). However, predicted changes in rainfall patterns (IPCC 2013) may also have potential consequences for phenology through delays in breeding in wet springs (Senapathi et al. 2011), or more directly for reproductive output (Siikamäki 1996; Franklin et al. 2000; Arlettaz et al. 2010). This is likely to occur via changes in prey activity (i.e., food availability; Avery and Krebs 1984), altered foraging patterns (Veistola et al. 1997; Dawson and Bortolotti 2000; Radford et al. 2001), and/or increased energy demands (Keller and van Noordwijk 1994; Siikamäki 1996; Veistola et al. 1997). Further, persistent or heavy rainfall may reduce juvenile growth rates (Siikamäki 1996; Veistola et al. 1997; Dawson and Bortolotti 2000) and increase offspring mortality (e.g., Siikamäki 1996; Dawson and Bortolotti 2000; Franklin et al. 2000; Rodríguez and Bustamante 2003; Arlettaz et al. 2010). Thus, predictions of how populations respond to on-going climate change will also require information on relationships between rainfall and vital rates for many species. One such potential group of species are insectivorous birds breeding in northern temperate regions, where mean precipitation and extreme precipitation events are predicted to increase with future climate change (IPCC 2013). Insectivores may be particularly affected by changing rainfall patterns because their main prey are less active, during adverse weather, resulting in reduced food availability (Avery and Krebs 1984).

In birds, the risk of rainfall-related nestling mortality could differ depending on the developmental stage of the nestlings (c.f. Low and Pärt 2009). This is because of a number of age-related changes that occur during nestling development that affect energy demand of altricial nestlings, including: (1) a change from an ectothermic state at hatching to an endothermic metabolism before fledging (Whittow 2000), (2) feathers grow and the surface area to volume ratio of individuals changes, reducing exposure and surface heat transfer (Whittow 2000), (3) body size and growth-rate changes during growth (e.g., Low et al. 2012), and (4) the time spent brooding nestlings decreases with nestling age (e.g., Conder 1989). As the direction of changes in energy demand is likely to differ for these factors – for example, chicks become more robust to exposure with time (i and ii above; and see Arlettaz et al. 2010), but potentially more susceptible to energy limitation with time (iii and iv above; and see Siikamäki 1996) – there are no simple predictions concerning nestling age and rainfall-related mortality.

Negative effects of rainfall on reproduction may not only be expressed as nestling mortality. Reduced foraging opportunities during rainfall (especially for insectivorous species) are likely to influence the condition of both young and their parents; thus, examinations of population impacts of rainfall patterns needs to consider long-term effects on individuals, such as reduced juvenile survival to the next year (Lindén et al. 1992; Naef-Daenzer et al. 2001; Low and Pärt 2009) and increased costs of reproduction in adults (Linden and Møller 1989; Stearns 1992). Weather effects on adult survival come from evolutionary studies during periods of adverse weather conditions (e.g., Boag and Grant 1981), or studies investigating effects of annual breeding season rainfall or large-scale weather patterns on adult survival, revealing negative effects of rainfall in some studies (Franklin et al. 2000; McDonald et al. 2004; Cowley and Siriwardena 2005), or no effects in other studies (Stokke et al. 2005; Robinson et al. 2008; Salewski et al. 2013). Clearly, if weather influences fledging success, recruitment success, and/or adult parental survival, estimates of effects need to consider multiple fitness components to avoid underestimating the impact of weather variables on population growth. At present, however, studies specifically linking rainfall during the nestling period with juvenile survival (e.g., recruitment success) and adult parental survival for species breeding in the northern temperate regions are largely lacking.

Here, we investigate the effects of rainfall during the nestling period on reproductive success and parental survival probability in a population of northern wheatears (*Oenanthe oenanthe*). First, we test whether rainfall is related to fledging success, recruitment success and apparent survival for male and female parents to the next year, while accounting for effects of temperature that otherwise may confound effects of rainfall. Second, we examine whether relationships between nestling mortality and rainfall are age dependent, that is, whether the strength of the effect of rainfall on fledging success, recruitment success, and apparent parental survival is limited to a specific period during nestling/fledgling development. Third, we investigate whether environmental or individual conditions such as habitat quality or timing of breeding may buffer individuals from effects of rainfall (Franklin et al. 2000). We expect individuals breeding in high-quality habitats to be less affected by rainfall than those in poorer quality habitats. Similarly, late breeding individuals may be more affected by rainfall than early breeders, as late breeders generally encounter deteriorating environmental conditions (e.g., reduced food availability; Perrins 1965; Öberg et al. 2014) or are poor quality individuals (de Forest and Gaston 1996; Morbey and Ydenberg 2000). Finally, we look at how parental nest feeding visits vary with rainfall as a possible mechanism explaining relationships between rainfall and fitness.

## Methods

### Study species, study area, and habitat characteristics

The northern wheatear (hereafter wheatear) is a cavity-breeding farmland bird (e.g., in stone piles at ground level, under roof tiles of buildings). Our study area is a 60 km^2^ heterogeneous agricultural landscape situated southeast of Uppsala in southern central Sweden (59°50′N, 17°50′E) and consists of 230 territory sites that have been occupied by wheatears at least once since 1993. Each year 120–180 pairs breed in the area. In a smaller core area (∼40 km^2^, 150 sites, 80–90 pairs per year), each territory site has been visited at least every third to 5th day throughout the breeding season to collect detailed data on demographic parameters.

The wheatear is a ground-foraging insectivore: preferring areas with short or sparse vegetation as their foraging strategies are adapted to such habitats (Conder 1989) and such habitats are related to high invertebrate availability (Tye 1992). Habitats with short ground vegetation layers (e.g., grazed pastures; hereafter short field layers) also have lower nest predation risk (Schneider et al. 2012), higher fledgling production (Pärt 2001a,b), and higher adult survival (Low et al. 2010) than habitats with tall and dense field layers (e.g., crops and unmanaged grasslands). The difference in adult survival is related to a habitat-specific difference in workload due to longer foraging flights in habitats with tall field layers (Low et al. 2010). Hence, height of the field layer is an important component of habitat quality (Arlt and Pärt 2007; Arlt et al. 2008).

Wheatears in this population migrate from sub-Saharan Africa and arrive at the study area in early to mid-April and the earliest individuals start laying eggs in the beginning of May. Hatching starts in the later part of May and both males and females participate in the care of nestlings. Young nestlings are altricial and dependent on brooding by the female to maintain body temperature. Nestlings were aged based on photos of known hatch-date nestlings of different ages, and hatching date was calculated from chick age. We recorded clutch size (number of eggs or number of chicks + unhatched eggs within 2 days of hatching), number of fledged young (number of ringed chicks minus number of dead chicks in the nest after fledging), and nest success (≥1 fledged young or intense warning calls from parents at the time of fledging at about 15 days of age). These proxies are generally accurate because most chick death comes from nest predation (Pärt 2001a). Nest predation almost always results in complete nest failure with removal of offspring from or destruction of the nest and parents will only warning call when offspring are still alive (Pärt 2001a; Schneider et al. 2012). Because we were interested in factors other than predation, we restricted our analyses to nests that were not preyed upon (i.e., successful nests and intact failed nests where the young had not been removed). Egg-laying date (i.e., the date the first egg was laid relative to 1 May) was estimated for all breeding attempts based on chick hatching dates (88% of all breeding attempts) and clutch size (either known, for 30% of all breeding attempts, or otherwise we assume a clutch size of six which is the mean for this population, see Öberg et al. 2014).

We ringed nestlings and adults with a unique combination of color-rings and a numbered metal ring (∼60% of adults from all established pairs, ∼90% nestlings that subsequently fledged). We aged adult birds as yearling or older based on their plumage characteristics (Pärt 2001a). Recruitment success and apparent parental survival was determined by re-sighting ringed birds in subsequent years. To minimize the potentially confounding effect of dispersal on recruitment and survival, we only estimated apparent parental survival and recruitment success for breeding attempts in the core part of the study area, using the surrounding 2 km area as a buffer zone for re-sighting of individuals (Arlt and Pärt 2008; Arlt et al. 2008; Low et al. 2010). Previous analyses have shown that restricting estimates to breeding attempts in the core zone, with subsequent re-sighting from the entire study area, results in unbiased estimates of adult and juvenile survival with respect to breeding habitat, sex, and age (Arlt et al. 2008; Low et al. 2010). Re-sighting probability for adults in this population is high (mean ± SE: males 0.98 ± 0.01; females 0.89 ± 0.03; Low et al. 2010), and because our survival estimates are unbiased with respect to individual and habitat covariates (Low et al. 2010), we estimated apparent survival estimates using return rates; this allowed us greater flexibility in a GLMM modeling framework (see below). Thus, while the relative effects of explanatory variables on survival will be largely unbiased, survival will be slightly underestimated because detection is not perfect.

We categorized territories according to their field layer height (FLH) and vegetation growth throughout the breeding season, as either short or tall (see also Pärt 2001a; Arlt and Pärt 2007). Field layers in short territories (farmyards and grazed pastures) were kept 5 cm or shorter throughout the breeding season while field layers in tall territories (crop fields, ungrazed pastures, and unmanaged grasslands) usually were short at territory establishment but grew to 15 cm or more at the time of chick rearing.

### Weather data

We obtained local weather data from the Ultuna Climate Station (59°82′N, 17°65′E; http://grodden.evp.slu.se/slu_klimat/index.html) recorded as daily amount of rainfall in millimeters (mm) and average daily temperature. Because we did not have data on hourly rainfall patterns for the whole study period, we could not investigate the details of how rain may be associated with reproductive performance and survival (e.g., several hours of continuous vs. short heavy rain). Instead, we used four daily based rainfall metrics measured during the nestling period (i.e., from day of hatching to day of fledging at 15 days of age for each individual nest): (1) total sum of rainfall (mm), (2) number of days with rain (i.e., >0 mm), because the duration of rainfall may be as important than the amount of rain (Dawson and Bortolotti 2000; Radford et al. 2001), (3) the longest sequence of consecutive days with rain, because the duration of rainfall may have larger impact if rain falls several days in a row and birds cannot recover condition, and (4) number of days with heavy rain (≥10 mm; after Skagen and Adams 2012).

### Nestling age

Because rainfall may affect reproduction and survival differently depending on the energetic requirements of the nestlings (see Introduction), we divided the nestling period into two periods based on growth curves for wheatear nestlings (Conder 1989) and expected development of thermoregulation for altricial species (Whittow 2000): (1) 0–7 days old; chicks are small but growing, ectothermic and highly dependent on brooding by the female, (2) 8–15 days old; increasing ability to thermoregulate and developing feathers covering most of the body after day 11 and onwards. After 8 days, nestlings likely reach the peak of their energy demand resulting from endothermic thermoregulation and growth and size (combination of high growth and larger body size requiring high energy for basal metabolism, see Low et al. 2012). Because young wheatears are also dependent on food provisioned by the parents during about 2 weeks after fledging, we also included a third period when fledged young were 16–25 days old.

### Statistical analysis

#### Reproductive success and survival

To test whether variation in fitness was related to variation in rainfall during individual nestling periods, we used data from 1994 to 2012 on fledging success (number of fledged young/clutch size from nests that were not preyed upon, *n* = 495), recruitment success (number of recruits/number of fledged young from successful nests, *n* = 665), and apparent parental survival (*n*_males_ = 714 and *n*_females_ = 741). The influence of the rainfall metrics on the three fitness components was analyzed using generalized linear mixed models in R (R Development Core Team 2012) with a binomial distribution (i.e., for fledging and recruitment success accounting for the number of trials per individual, clutch size or number fledged per nest), and logit link (function “lmer” in the package lme4; Bates et al. 2012). We used this approach for analyzing fledging success rather than a nest survival analysis (e.g., Mayfield 1975) because we rarely had accurate data on the timing of individual offspring death within the nest, and most complete nest failures were removed from our analyses because they resulted from predation (see also Schneider et al. 2012; Öberg et al. 2014). Analyses were implemented in an information-theoretic framework (Burnham and Anderson 2002). We used a two-step approach for model selection to reduce model complexity. First, we identified which of the four different rain variables best explained variation in the fitness component of interest (step 1). The resulting highest ranked model was then used as a basis for examining the role of additional covariates, including disentangling the potentially confounding effects of temperature (step 2).

##### Step 1 – Assessing rainfall-fitness correlations

For each fitness component, we created a candidate model set, where we varied rainfall variables while holding constant variables known to be important in influencing each respective fitness component (based on Öberg et al. 2014 where relative variable importance weights were >0.60), which included: (1) for fledging success – field layer height (short/tall), female age (yearling/older), egg-laying date (the date the first egg was laid relative to 1 May), and an age*egg-laying date interaction, (2) for recruitment success – field layer height, female age, nest type (roof vs. ground), and egg-laying date, and (3) for female and male parental survival – field layer height, nest success (successful/failed), and egg-laying date. Although egg-laying date does not appear to influence adult survival (Öberg et al. 2014), we included this factor in the base models because any seasonal pattern in rainfall may hide a potential relationship between survival and egg-laying date. Similarly, age did not appear to influence adult survival (Low et al. 2010), but due to a higher proportion of young breeders in tall habitats (tall vs. short; females: 48 vs. 35%, *χ*^2^ = 11.7, *P* < 0.001; males: 39% vs. 26%, *χ*^2^ = 12.2, *P* < 0.001), we included age to avoid any confounding effects of this variable on parental survival. For both fledging and recruitment success, female age was unknown in ∼20% of the breeding attempts, so we used the known male age in these cases because male and female age is highly correlated in this population (likelihood ratio, *χ*^2^ = 79.44, *P* < 0.001, *n* = 820; see also Öberg et al. 2014). For female parental survival analyses, we only used females of known age. In all models, we included year and individual identity as random effects; except for the fledging success analysis where the individual random effect resulted in some models not converging and so it was excluded. This omission was unlikely to have influenced our overall results, as the term did not improve AIC (Akaike's information criterion) in less complex fledging success models where convergence occurred.

Using the variables known to influence each respective fitness component as a base, we created models for each rainfall measure (see above) during periods of different nestling ages: the entire nestling period (when chicks were 0–15 days of age), the first half of the nestling period (0–7 days), and the second half of the nestling period (8–15 days); for recruitment success and parental survival, there was one additional model for each rainfall measure during the early postfledging period (16–25 days). Thus, the candidate model set contained 13 models for fledging success (base + 4 rain variables x 3 periods) and 17 models for recruitment success and parental survival (base + 4 rain metrics x 4 periods). The initial analyses included the rainfall measure only as a linear term, as visualization of the raw data showed no obvious nonlinear relationships between rainfall and the fitness components of interest (not shown). All models in the candidate set were then ranked by AIC, with the highest ranked model (smallest AIC; Table S1) used as the base model for the next step in the analyses of each fitness component providing the addition of a rainfall variable improved AIC by >2 (Burnham and Anderson 2002). For female parental survival, the effect of rainfall on model fit was extremely weak (ΔAIC < 2; Table S1); thus, we concluded that there was little support for an effect of rainfall on female parental survival and did not perform any further analysis on this fitness component.

##### Step 2 – Disentangling temperature and rainfall and assessing rainfall–covariate interactions

We built new candidate model sets for each fitness component based on the highest ranked model from step 1. First, to account for potential effects of temperature on variation in fitness components, we included mean temperature during the same time period as for the rain variable. Temperature and rainfall were correlated but Pearson's correlation coefficients never exceeded 0.4, and the variance inflation factor (VIF) for our fixed effects was always <1.7 (a VIF > 2 suggests problems with collinearity; Zuur et al. 2009). Second, we included an interaction between field layer height and rainfall to investigate whether habitat quality may buffer individuals from effects of rainfall. Third, we included an interaction between egg-laying date and rainfall to investigate whether rainfall-related effects on fitness differ for early and late breeding individuals. Finally, we included the interaction between number of fledged young and rainfall in models of male parental survival. If rainfall effects on survival are mediated through an increase in effort, individuals with many young (i.e., higher effort) should be more vulnerable to rainfall. Including all combinations of those additional covariates resulted in candidate sets of nine models for fledging and recruitment success, and 17 models for male parental survival. Models were compared based on AIC values and AIC weights (Burnham and Anderson 2002). As a validation for the model selection in step 1, we then reran all of the top-ranked models in step 2 with the different rainfall parameters from step 1; in no cases did the model fit improve with other rainfall data, supporting the step 1 model selection procedure. We then also checked our assumption that the relationship between rainfall and fitness was linear by comparing the full model with a model that included a quadratic rainfall term; with the exception of fledging success, the quadratic term did not improve model fit. We report the quadratic rainfall effect for fledging success below.

#### Nest visitation rates

The number of feeding visits per hour was measured for 39 nests between 2007 and 2010 by data-loggers fitted into the entrance hole of the nest, recording all movements by parents in and out of the nest (for further details on the method, see Low et al. 2008). We derived a base model from Low et al. (2008) who found that nest visitation rates in this population depend on time of day (linked to prey activity) and ambient temperature. We included hour and its quadratic term, but as the mean hourly temperature closely follows the diurnal nest visitation pattern (Low et al. 2008), we did not include mean hourly temperature in the base model. We included nestling age and its quadratic term in the base model to account for age-dependent feeding visit rates. Because data were over-dispersed (over-dispersion = 7.5), we included an id-variable containing unique numbers for each observation (i.e., number of visits/hour) as a random factor. We also included nest identity as a random factor to account for dependencies within nests. We used generalized linear mixed models with a Poisson error distribution and a log link function. To investigate whether rainfall across the entire day (i.e., daily rainfall) or rainfall during preceding days affected nest visitation rates, we set up five candidate models including the base model (see above), and models including separately daily rainfall, amount of rainfall one the day before the day of visitation, over the 2 days before, and over the 3 days before. The latter three models also included daily rainfall on the day of visitation. We assessed these by AIC values and AIC weights.

## Results

### Rainfall and fledging success

For the initial rainfall-fitness analysis (i.e., step 1), there was strong support for reduced reproductive output with increased rainfall during the nestling period (Table S1). The rain variable that explained most of the variation in fledging success was number of days with ≥10 mm rain. By comparing models with number of days with ≥10 mm rain during: (1) the entire nestling period (NumDays10_NestFull_), (2) the first half of the nestling period (NumDays10_Nest1_), and (3) the second half of the nestling period (NumDays10_Nest2_), it was clear that nestling sensitivity to rainfall is almost exclusive to the second half of the nestling period (Table S1).

**Figure 1:**
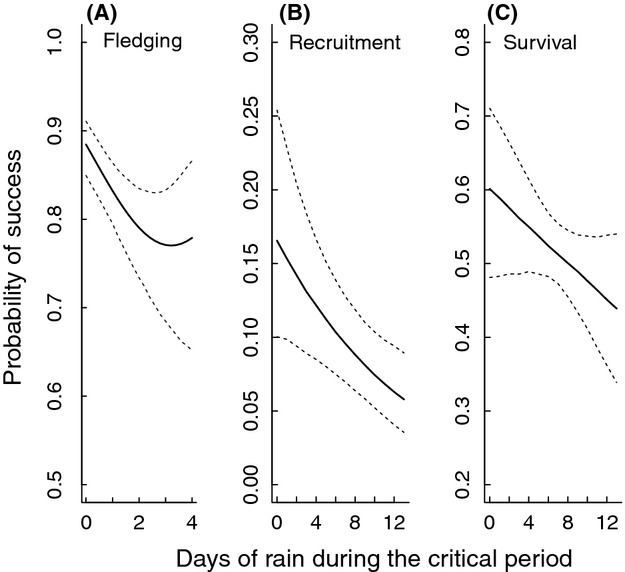
Relationship between (A) fledging success (number of fledged young per egg laid) and number of days with ≥10 mm of rain during the second half of the nestling period, (B) recruitment success (number of recruits per fledged young) and number of days with rain during the entire nestling period, and (C) male parental survival and number of days with rain during the entire nestling period. Lines are model predictions with their associated 95% CIs from the respective highest ranked model in Table1. Explanatory variables other than the rainfall variables were fixed at their mean values.

**Figure 2:**
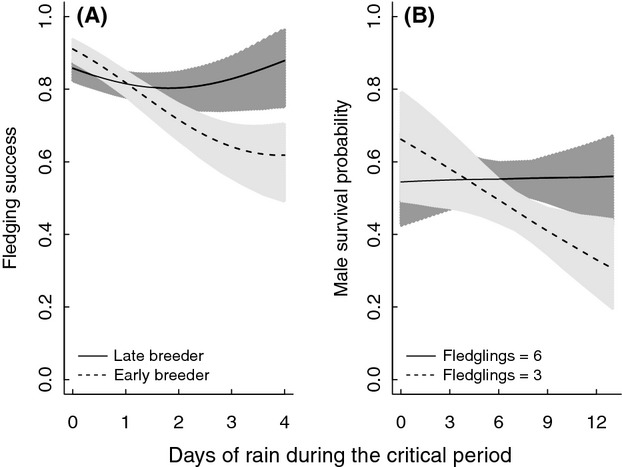
Interactions between (A) egg-laying date (ELD) and the number of days with ≥10 mm of rain during the second half of the nestling period on fledging success (number of fledged young per egg laid), and (B) the number of chicks fledged and the number of days with rain during the entire nestling period on male parental survival. For fledging success, the solid line with dark gray shading represents early breeders (20% quantile; ELD = 10); the dashed line with light gray shading is later breeders (80% quantile; ELD = 21). For male parental survival, the solid line is for nests that fledged a large number of young (80% quantile; fledglings = 6), while the dashed line is for nests with fewer offspring fledged (20% quantile; fledglings = 3). Lines are model predictions with their associated 95% CIs from the respective highest ranked model in Table1. Other explanatory variables were fixed at their mean values.

When considering additional covariates (i.e., including average daily temperature and interactions, see “step 2” in methods), there was support for an effect of temperature on fledging success, as models containing this variable always had more support than equivalent models without it (Table1). Fledging success was higher when average temperature was warmer during the second half of the nestling period (Table2), with the number of days with ≥10 mm rain continuing to be an important determinant of fledging success when temperature was included (Table1; Fig.1A). Furthermore, there was an interaction between rainfall and egg-laying date on fledging success (Tables1 and 2); the rainfall effect was stronger for early than late breeding birds (Fig.2A). By including a quadratic term for the rainfall variable, there was clear support for the relationship between fledging success and rainfall during the second half of the nestling period being nonlinear (Tables1 and 2; Fig.2A); rainfall effects on fitness appeared to plateau at ∼3 days. There was no clear interactive effect between rainfall and field layer height on fledging success as models containing this interaction always had less support than the equivalent model without it (Table1).

**Table 1:** AIC-ranked candidate models relating rainfall variables and other covariates to fledging success, recruitment success, and male parental survival

Model structure	K	AIC	ΔAIC	*w*_*i*_
Fledging success				
Base + ELD x rain + rain^2^ + temp	11	933.11	0	0.96
Base + ELD x rain + temp	10	940.02	6.91	0.02
Base + ELD x rain + rain^2^	10	940.23	7.12	0.01
Base + rain + temp	9	940.64	7.53	0.01
Base + ELD x rain + FLH x rain + temp	11	941.16	8.05	0.00
Base + FLH x rain + temp	10	941.20	8.09	0.00
Base + rain + rain^2^	9	947.61	14.5	0.00
Base + temp	8	949.56	16.45	0.00
Base + ELD x rain	9	950.60	17.49	0.00
Base + ELD x rain + FLH x rain	10	951.32	18.21	0.00
Base + FLH x rain	9	953.26	20.15	0.00
Base + rain	8	953.63	20.52	0.00
Base	7	959.99	26.88	0.00
Recruitment success				
Base + rain	8	838.23	0.00	0.31
Base + ELD x rain	9	839.25	1.02	0.18
Base + rain + temp	9	840.06	1.83	0.12
Base + FLH x rain	9	840.12	1.89	0.11
Base + ELD x rain + temp	10	841.04	2.81	0.08
Base + ELD x rain + FLH x rain	10	841.20	2.97	0.07
Base + FLH x rain + temp	10	841.94	3.71	0.05
Base + temp	8	842.20	3.97	0.04
Base + ELD x rain + FLH x rain + temp	11	842.99	4.76	0.03
Base	7	844.11	5.88	0.01
Male parental survival				
Base + fledgling x rain	9	834.36	0.00	0.28
Base + fledgling x rain + ELD x rain	10	835.69	1.33	0.15
Base + fledgling x rain + FLH x rain	10	835.99	1.63	0.13
Base + fledgling x rain + temp	10	836.33	1.97	0.11
Base + fledgling x rain + FLH x rain + ELD x rain	11	837.38	3.02	0.06
Base + fledgling x rain + ELD x rain + temp	11	837.57	3.21	0.06
Base + fledgling x rain + FLH x rain + temp	11	837.96	3.60	0.05
Base + rain	8	838.60	4.24	0.03
Base + ELD x rain	9	838.94	4.58	0.03
Base + fledgling x rain + FLH x rain + ELD x rain + temp	12	839.27	4.91	0.02
Base + temp	8	839.64	5.28	0.02
Base + FLH x rain	9	839.82	5.46	0.02
Base + FLH x rain + ELD x rain	10	840.35	5.99	0.01
Base + rain + temp	9	840.60	6.24	0.01
Base + ELD x rain + temp	10	840.84	6.48	0.01
Base	7	841.12	6.76	0.01
Base + FLH x rain + temp	10	841.81	7.45	0.01
Base + FLH x rain + ELD x rain + temp	11	842.25	7.89	0.00

The base models were GLMMs that included the bird's age, nest location, egg-laying date (ELD), field layer height (FLH), with year and individual as random effects (see Methods). The rainfall variables (rain) were those from the highest ranked model for each fitness component in Table S1 (fledging success = number of days with ≥10 mm of rain during the second half of the nestling period; recruitment and male survival = number of days with rain during the nestling period), with rain^2^ representing the quadratic term. Mean temperature (temp) was calculated from the same period as the rainfall variable. Additive effects are (+), interactions (x) and number of fledglings (fledgling); K = number of parameters, ΔAIC = difference in AIC relative to the highest ranked model, *w*_*i*_* *= AIC weight of the model. Pseudo-*R*^2^ values (Nakagawa and Schielzeth 2013) for key models are given in Table S2.

**Table 2:** Model parameter estimates (±SE) from the highest ranked model from Table1 for fledging success, recruitment success, and male parental survival

Variables	Fledging success	Recruitment success	Male parental survival
Intercept	0.57 ± 0.68	−1.380 ± 0.312	1.027 ± 0.669
AgeF_Yearling_	r0.556 ± 0.321	0.199 ± 0.152	–
AgeM_Yearling_	–	–	−0.250 ± 0.192
FLH_Tall_	−0.547 ± 0.109	−0.471 ± 0.151	−0.052 ± 0.179
ELD	−0.034 ± 0.013	−0.012 ± 0.014	0.011 ± 0.014
Nest type	–	−0.518 ± 0.219	–
Rain	−1.41 ± 0.346	−0.103 ± 0.036	−0.230 ± 0.085
Rain^2^	0.120 ± 0.038	–	–
Fledglings	–	–	−0.151 ± 0.118
Temperature	0.154 ± 0.045	–	–
AgeF_Yearling_ x ELD	−0.044 ± 0.017	–	–
ELD x Rain	0.046 ± 0.014	–	–
Fledglings x Rain	–	–	0.039 ± 0.016

Estimates are from binomial GLMMs (logit link) with year and individual as random effects. Variables include male or female age (AgeM or AgeF, respectively: yearling vs. older), field layer height (FLH; short vs. tall), egg-laying date (ELD relative to May 1st), nest type (ground vs. roof), rain (fledging success = number of days with ≥10 mm of rain during the second half of the nestling period; recruitment and male survival = number of days with rain during the nestling period), number of nestlings fledged (fledglings), and mean temperature during the critical rain period. Interactions between variables are indicated by “x”.

**Table 3:** Model parameter estimates (±SE) for the highest ranked model (with Poisson error term and log link) for hourly nest visitation rates (Table S1)

Variables	Estimate ± SE
(Intercept)	1.150 ± 0.074
Hour	0.186 ± 0.006
Hour^2^	−0.008 ± 0.001
Age	0.204 ± 0.008
Age^2^	−0.012 ± 0.001
Rain	−0.014 ± 0.002
3 day before	−0.004 ± 0.001

Variables are hour of the day, age of the chicks (days since hatching), rain (in mm during that hour), and the amount of rain in the preceding 3-day period (3 day before). The model includes nest and observational level random effects.

### Rainfall and recruitment

Variation in recruitment success was best explained by the number of days with rain during the entire nestling period (NumDays_NestFull_; Table S1); this variable had substantially more support than the base model and models with other rainfall variables. When dividing the entire nestling period into two parts, there was more support for an effect of rain on juvenile survival during the early nestling period (NumDays_Nest1_) than during the second half (NumDays_Nest2_; Table S1).

The step 2 covariate analysis revealed only weak support for an effect of average temperature during the nestling stage, while the number of days with rain was still negatively related to juvenile survival (Tables1 and 2; Fig.1B). There was no clear support for any interactive effects between rainfall and field layer height or egg-laying date (Table1).

### Rainfall and subsequent parental survival

The step 1 rainfall analysis on male parental survival to the next year showed support for declining survival as number of days with rain increased during the nestling stage (NumDays_NestFull_; Table S1; Fig.1C). This model was closely followed by the equivalent model containing rainfall during the first part of the nestling period (NumDays_Nest1_), suggesting that much of the rainfall effect on male survival is during the earlier nestling period (Table S1). For female parental survival, no model containing rainfall substantially improved model fit (ΔAIC > 2) as compared to the base model (Table S1).

For the step 2 covariate analyses, there was little support for an effect of average temperature on male parental survival as models containing temperature always had less support than the equivalent model without it (Table1). However, there was substantial support for an interactive effect between rain and number of fledged young on male parental survival (Tables1 and 2). In contrast to our prediction, for males who fledged a smaller number of offspring, there was a steeper decline in apparent survival with increasing numbers of days with rainfall, than for those with more fledglings (Table2; Fig.2B). There was little support for interactive effects between rainfall and field layer height or egg-laying date on male parental survival (Table1).

### Visitation rates

**Figure 3:**
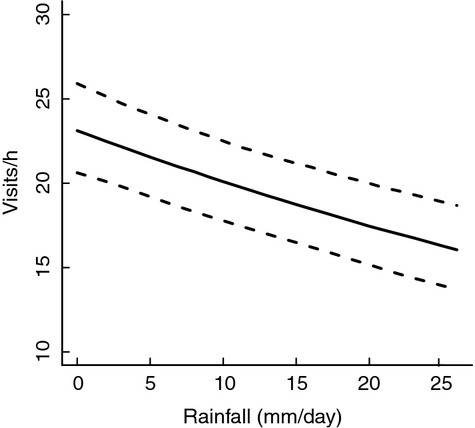
The relationship between the number of visits per hour and daily amount of rainfall during day of visitation. Lines are model predictions and their 95% CIs from the top-ranked model (Table S1) when all explanatory variables other than daily rainfall (i.e., hour, hour^2^, chick age, chick age^2^, and prior rain) were fixed at their mean values.

Daily rainfall was important in explaining daily variation in parental visitation rates to nestlings, as increasing the amount of daily rainfall reduced average hourly visitation rates (Tables S1 and 3; Fig.3). This negative effect was further enhanced by the amount of rainfall over 2 or 3 days preceding the day of visitation (Tables S1 and 3).

## Discussion

Our detailed individual-based data enabled us to investigate how different demographic components were related to variation in rainfall (independent of temperature) during nestling care. Rainfall during the nestling period not only reduced fledging success, but also recruitment success and apparent male parental survival (Fig.1), although there was no clear effect on female parental survival. The amount of rainfall reduced visitation rates (Fig.3), which suggests chicks received less food during rain events and thus potentially explains the reduced fledging success and subsequent juvenile survival (i.e., recruitment success). Our results confirm previous studies showing the negative effects of rainfall on the production of fledglings (Siikamäki 1996; Dawson and Bortolotti 2000; Franklin et al. 2000; Rodríguez and Bustamante 2003; Arlettaz et al. 2010). But more importantly, we show that effects of rainfall during the nestling stage may have long-term effects on fitness such as recruitment and parental survival probabilities. We also found the effect of rainfall on reproductive performance to be stronger for early as compared to late breeders (Fig.2A), whereas there was no support for an interaction between field layer height (reflecting territory quality) and rainfall on investigated components of fitness.

### Visitation rates

Daily rainfall reduced parental visitation rates by ∼22% during days with heavy rain (e.g., >20 mm; Fig.3). Similarly, other studies have shown nestling provisioning rates to decline with increasing amount of rain (e.g., Dawson and Bortolotti 2000; Radford et al. 2001; Geiser et al. 2008; Arlettaz et al. 2010). Visitation rates may be reduced by rainfall either through increased time spent brooding to compensate for increased thermoregulatory demands of the young (Radford et al. 2001) or through the commonly suggested reduction in prey availability during rainfall (Avery and Krebs 1984; Veistola et al. 1997). Lower visitation rates may in turn result in less food provisioned to chicks. Changes in visitation rates could be compensated for by changes in load size (i.e., the amount of food delivered per nest visit, Grieco 2002) although some studies suggest that also load size may decrease when it is raining (Dawson and Bortolotti 2000; Arlettaz et al. 2010). The combined results of reduced food visitation rates and reduced reproductive performance strongly suggest that rainfall affects the amount and possibly quality of food (Arlettaz et al. 2010) provided to nestlings.

### Fledging and recruitment success

The negative effect of rainfall on fledging success was stronger in the later part of the nestling period when the overall energy demands of the nestlings are expected to be the highest. This suggests that food shortages during the period with highest net energy demand for offspring (because of growth, body size, and endothermic metabolism) may reduce their condition below a threshold from which they cannot recover. Younger nestlings, on the other hand, are ectothermic and hence may be able to reduce their metabolism to low levels during periods of adverse weather conditions. Furthermore, young nestlings are often brooded by the female (Conder 1989), and during poor weather conditions, females may increase their time spent brooding to protect the young (Radford et al. 2001; own observations).

By contrast, recruitment success was related to the number of days of rainfall during the first half of the nestling period. Rainfall effects may not only increase the risk of mortality for nestlings but may also reduce growth rates (Conder 1989; Keller and van Noordwijk 1994; Siikamäki 1996; Veistola et al. 1997; Dawson and Bortolotti 2000). Fledgling body condition has been shown to strongly affect probability of juvenile survival to the next year (Sullivan 1989; Lindén et al. 1992; Naef-Daenzer et al. 2001; Low and Pärt 2009). As the ability to compensate reduced growth during adverse weather may be lower for young as compared to old nestlings (Geiser et al. 2008; but see Siikamäki 1996), it is possible that effects of rain during the early nestling stage may be manifested as reduced survival after fledging.

Our results also indicate a stronger effect of rainfall for nests hatched early than late in the breeding season (Fig.2A). Fledging and recruitment success is generally lower for nests hatched late in the breeding season (Öberg et al. 2014) and thus, effects further reducing reproductive performance may be more difficult to detect (lower statistical power). However, nestlings hatched early in the season may also be more sensitive to rainfall. Early breeding individuals produce on average larger clutches and have larger broods than late breeders (Öberg et al. 2014), potentially requiring an effort beyond the parent's capacity when feeding young during poor environmental conditions thus causing brood reduction through nestling mortality (Siikamäki 1996). If this is true, difference in fitness between early and late breeders may disappear in years with heavy rain early in the season, reducing the advantages of early breeding.

### Parental survival

For parents, the energetically most demanding period during breeding is when feeding nestlings (Moreno and Hillström 1992; Newton 1998) and any factor affecting nestling food provisioning effort may thus affect subsequent parental survival. As prey items become more difficult to find, parents may be forced to increase the time spent foraging and cover greater distances during foraging bouts to avoid starvation of their chicks (Low et al. 2010), and by increasing investment in their offspring, they may also compromise their own needs (Nilsson 2002) and get less food for themselves (Markman et al. 2002).

Our analyses suggest that parental survival in males was more sensitive than females to the effects of increased rainfall during the period of nestling care. Radford et al. (2001) showed that female great tits (*Parus major*) increased the time spent brooding during rainfall and that this increase accounted for the entire reduction in visitation rates. In the wheatear, only females brood (Conder 1989) and wheatear females are likely to reduce visitation rates due to increased brooding during adverse weather conditions. This could explain why female parental survival was not reduced when there was more rainfall and at the same time explain why male parental survival should be linked to number of days with rain especially during the first nestling stage (Tables1 and 2). As female brooding of nestlings takes place mainly during the early nestling period (chick age ≤5 days; Conder 1989), increased brooding due to adverse weather may force males to increase their effort to compensate for the reduced provisioning rates of females.

The interaction between rainfall and number of fledged young (Fig.2B) showed that the negative effect of rainfall on male parental survival increased during complete nest failure as compared to successfully fledging young. This was in contrast to our expectation based on the assumption that effort increases with the number of fledglings produced. However, male reproductive effort may be more closely linked to adverse weather conditions than to the number of fledglings produced, especially if the conditions are so bad that the brood is largely reduced or the breeding attempt fails completely. This is because males not only may have to compensate for female reduction in nestling food provisioning; they have to do that in the worst conditions. Thus, the relationship between male parental survival and the number of offspring fledged may largely arise from the fact that high-quality males in good condition can fledge offspring and subsequently survive, while males in poorer condition are more likely to fail at both tasks. Breeding failures were linked to amount of rainfall (data not shown) and a combination of rain and failure likely reflected extremely poor conditions for foraging and nestling food provisioning. We suggest that the effects of rain on male parental survival could be driven by a sexual difference in parental duties during adverse weather conditions. Detailed data on male and female food provisioning rates in relation to rainfall and reproductive parameters could test this hypothesis.

This study adds to our knowledge of weather effects on individual fitness by showing that (1) rain during the nestling stage not only relates to fledging success but may also have long-term effects on subsequent juvenile (as measured by recruitment success) and parental survival, (2) rainfall may have different effects on reproductive output and survival depending on when in the nestling stage it rains, and (3) the effect of rainfall is more marked among early than late breeders. In the northern temperate region, climate change scenarios indicate an increasing number of days of heavy rain, greater weather variability, and occurrence of extreme weather events in the future (IPCC 2013). Our results stress that knowledge of weather effects other than temperature on multiple vital rates are crucial if we want to understand or predict population responses to future climate change.

## Supporting Information

**Table S1.**Candidate set of AIC-ranked models relating rainfall variables to fledging & recruitment success, parental survival and visitation rates. **Table S2.** Marginal and conditional pseudo-*R*^2^ values for key models in Table1.
