# Supplementary material for: Rainfall during parental care reduces reproductive and survival components of fitness in a passerine bird
Source: Ecol Evol. 2014 Dec 24;5(2):345–56. doi: 10.1002/ece3.1345 (PMC4314267; doi:10.1002/ece3.1345)
Supplement: Supplementary file 1 [file ece30005-0345-sd1.docx]

**Rainfall during parental care reduces reproductive and survival components of fitness in a passerine bird**

Meit Öberg*, Debora Arlt, Tomas Pärt, Ane T. Laugen, Sönke Eggers and Matthew Low

Online Resource

**Tables S1 & S2**

**Table S1.** Full candidate set of AIC-ranked models relating rainfall variables to fledging success, recruitment success & adult parental survival and visitation rates. The base model is indicated in bold. Variables for reproductive success and survival models: AgeF=female age (yearling/older), ELD=egg-laying date (relative to May 1^st^ each year), FLH=field layer height (short/tall), Nest=nest type (roof/ground), SumRain=sum of rainfall (mm), NumDays=number of days with > 0 mm of rainfall, RainDays=number of consecutive days with > 0 mm rainfall and NumDays10=number of days with ≥ 10 mm of rainfall. Indices for rain variables during either the full nestling period (0-15 days) or different nestling or early fledgling stages: _NestFull_ (0-15 days), _Nest1_ (0-7 days), _Nest2_ (8-15 days) and _Nest3_ (16-25 days). Variables for nest visitation models: H = hour of the day, age = chick age, rain= daily amount of rainfall (mm), 3day = amount of rainfall in the three days before, 2day = amount of rainfall in the two days before, 1day = amount of rainfall in the day before. K= number of parameters, ΔAIC = difference in AIC relative to the best model, *w_i_*=AIC weight of the model.

| Model structure | K | AIC | ΔAIC | *w_i_* |
| --- | --- | --- | --- | --- |
| *Fledging success* |  |  |  |  |
| AgeF x ELD + FLH + NumDays10_Nest2_ | 8 | 953.63 | 0.00 | 0.328 |
| AgeF x ELD + FLH + NumDays10_NestFull_ | 8 | 953.94 | 0.31 | 0.281 |
| AgeF x ELD + FLH + RainDays_Nest1_ | 8 | 954.90 | 1.27 | 0.174 |
| AgeF x ELD + FLH + SumRain_Nest2_ | 8 | 955.95 | 2.32 | 0.103 |
| AgeF x ELD + FLH + SumRain_NestFull_ | 8 | 958.14 | 4.51 | 0.034 |
| AgeF x ELD + FLH + NumDays_Nest1_ | 8 | 959.67 | 6.04 | 0.016 |
| AgeF x ELD + FLH + RainDays_NestFull_ | 8 | 959.91 | 6.28 | 0.014 |
| AgeF x ELD + FLH | **7** | **959.99** | **6.36** | **0.014** |
| AgeF x ELD + FLH + RainDays_Nest2_ | 8 | 960.54 | 6.91 | 0.010 |
| AgeF x ELD + FLH + NumDays_Nest2_ | 8 | 960.56 | 6.93 | 0.010 |
| AgeF x ELD + FLH + SumRain_Nest1_ | 8 | 961.65 | 8.02 | 0.006 |
| AgeF x ELD + FLH + NumDays_NestFull_ | 8 | 961.89 | 8.26 | 0.005 |
| AgeF x ELD + FLH + NumDays10_Nest1_ | 8 | 961.95 | 8.32 | 0.005 |
| *Recruitment success* |  |  |  |  |
| AgeF + FLH + ELD + Nest + NumDays_NestFull_ | 8 | 838.23 | 0.00 | 0.579 |
| AgeF + FLH + ELD + Nest + NumDays_Nest1_ | 8 | 840.85 | 2.62 | 0.156 |
| AgeF + FLH + ELD + Nest + NumDays10_Nest3_ | 8 | 844.01 | 5.78 | 0.032 |
| AgeF + FLH + ELD + Nest | **7** | **844.11** | **5.88** | **0.031** |
| AgeF + FLH + ELD + Nest + NumDays_Nest3_ | 8 | 844.47 | 6.24 | 0.026 |
| AgeF + FLH + ELD + Nest + NumDays_Nest2_ | 8 | 844.88 | 6.65 | 0.021 |
| AgeF + FLH + ELD + Nest + SumRain _Nest3_ | 8 | 845.16 | 6.93 | 0.018 |
| AgeF + FLH + ELD + Nest + RainDays_Nest3_ | 8 | 845.19 | 6.96 | 0.018 |
| AgeF + FLH + ELD + Nest + SumRain_Nest1_ | 8 | 845.34 | 7.11 | 0.017 |
| AgeF + FLH + ELD + Nest + NumDays10_NestFull_ | 8 | 845.67 | 7.44 | 0.014 |
| AgeF + FLH + ELD + Nest + SumRain_Nest2_ | 8 | 845.68 | 7.45 | 0.014 |
| AgeF + FLH + ELD + Nest + NumDays10_Nest2_ | 8 | 845.70 | 7.47 | 0.014 |
| AgeF + FLH + ELD + Nest + RainDays_NestFull_ | 8 | 845.72 | 7.49 | 0.014 |
| AgeF + FLH + ELD + Nest + RainDays_Nest2_ | 8 | 845.95 | 7.72 | 0.012 |
| AgeF + FLH + ELD + Nest + RainDays_Nest1_ | 8 | 845.98 | 7.75 | 0.012 |
| AgeF + FLH + ELD + Nest + SumRain_NestFull_ | 8 | 846.04 | 7.81 | 0.012 |
| AgeF + FLH + ELD + Nest + NumDays10_Nest1_ | 8 | 846.10 | 7.87 | 0.011 |
| *Male parental survival* |  |  |  |  |
| AgeM + FLH + ELD + NS + NumDays_NestFull_ | 8 | 991.71 | 0.00 | 0.243 |
| AgeM + FLH + ELD + NS + NumDays_Nest1_ | 8 | 992.18 | 0.47 | 0.192 |
| AgeM + FLH + ELD + NS + RainDays_Nest1_ | 8 | 993.83 | 2.12 | 0.084 |
| AgeM + FLH + ELD + NS | **7** | **994.23** | **2.52** | **0.069** |
| AgeM + FLH + ELD + NS + SumRain_NestFull_ | 8 | 994.93 | 3.22 | 0.049 |
| AgeM + FLH + ELD + NS + SumRain_Nest1_ | 8 | 995.42 | 3.71 | 0.038 |
| AgeM + FLH + ELD + NS + NumDays_Nest2_ | 8 | 995.47 | 3.76 | 0.037 |
| AgeM + FLH + ELD + NS + SumRain_Nest3_ | 8 | 995.47 | 3.76 | 0.037 |
| AgeM + FLH + ELD + NS + NumDays_Nest3_ | 8 | 995.60 | 3.89 | 0.035 |
| AgeM + FLH + ELD + NS + SumRain_Nest2_ | 8 | 995.75 | 4.04 | 0.032 |
| AgeM + FLH + ELD + NS + NumDays10_NestFull_ | 8 | 996.02 | 4.31 | 0.028 |
| AgeM + FLH + ELD + NS + NumDays10_Nest2_ | 8 | 996.06 | 4.35 | 0.028 |
| AgeM + FLH + ELD + NS + NumDays10_Nest1_ | 8 | 996.17 | 4.46 | 0.026 |
| AgeM + FLH + ELD + NS + RainDays_Nest2_ | 8 | 996.19 | 4.48 | 0.026 |
| AgeM + FLH + ELD + NS + RainDays_NestFull_ | 8 | 996.19 | 4.48 | 0.026 |
| AgeM + FLH + ELD + NS + RainDays_Nest3_ | 8 | 996.21 | 4.50 | 0.026 |
| AgeM + FLH + ELD + NS + NumDays10_Nest3_ | 8 | 996.22 | 4.51 | 0.025 |
| *Female parental survival* |  |  |  |  |
| AgeF + FLH + ELD + NS + NumDays_Nest2_ | 8 | 1026.8 | 0.00 | 0.158 |
| AgeF + FLH + ELD + NS | **7** | **1027.6** | **0.80** | **0.106** |
| AgeF + FLH + ELD + NS + RainDays_Nest2_ | 8 | 1027.8 | 1.00 | 0.096 |
| AgeF + FLH + ELD + NS + RainDays_Nest1_ | 8 | 1028.2 | 1.40 | 0.079 |
| AgeF + FLH + ELD + NS + SumRain_Nest1_ | 8 | 1028.8 | 2.00 | 0.058 |
| AgeF + FLH + ELD + NS + NumDays_Nest1_ | 8 | 1029.0 | 2.20 | 0.053 |
| AgeF + FLH + ELD + NS + NumDays_NestFull_ | 8 | 1029.3 | 2.50 | 0.045 |
| AgeF + FLH + ELD + NS + NumDays10_NestFull_ | 8 | 1029.4 | 2.60 | 0.043 |
| AgeF + FLH + ELD + NS + SumRain_NestFull_ | 8 | 1029.5 | 2.70 | 0.041 |
| AgeF + FLH + ELD + NS + SumRain_Nest3_ | 8 | 1029.5 | 2.70 | 0.041 |
| AgeF + FLH + ELD + NS + NumDays_Nest3_ | 8 | 1029.5 | 2.70 | 0.041 |
| AgeF + FLH + ELD + NS + NumDays10_Nest2_ | 8 | 1029.5 | 2.70 | 0.041 |
| AgeF + FLH + ELD + NS + RainDays_Nest3_ | 8 | 1029.5 | 2.70 | 0.041 |
| AgeF + FLH + ELD + NS + NumDays10_Nest1_ | 8 | 1029.6 | 2.80 | 0.039 |
| AgeF + FLH + ELD + NS + SumRain_Nest2_ | 8 | 1029.6 | 2.80 | 0.039 |
| AgeF + FLH + ELD + NS + RainDays_NestFull_ | 8 | 1029.6 | 2.80 | 0.039 |
| AgeF + FLH + ELD + NS + NumDays10_Nest3_ | 8 | 1029.6 | 2.80 | 0.039 |
| *Nest visitation rate* |  |  |  |  |
| H + H^2^ + age + age^2^ + rain + 3day | 9 | 18676 | 0.00 | 0.726 |
| H + H^2^ + age + age^2^ + rain + 2day | 9 | 18678 | 2.00 | 0.267 |
| H + H^2^ + age + age^2^ + rain | 8 | 18686 | 10.00 | 0.005 |
| H + H^2^ + age + age^2^ + rain + 1day | 9 | 18688 | 12.00 | 0.002 |
| H + H^2^ + age + age^2^ | **7** | **18719** | **43.00** | **0.000** |

**Table S2.** Pseudo-R-squared values from the base models, the base + rainfall models and the highest-ranked models in Table 1 (see Table 1 in the main paper for explanations of variables and models). Both marginal and conditional R-squared values are given; these were calculated in the R package ‘MuMIn’ (Barton 2013) based on the paper by Nakagawa & Schielzeth (2013).

| Model structure | AIC | Marginal R^2^ | Conditional R^2^ |
| --- | --- | --- | --- |
| *Fledging success* |  |  |  |
| Base + ELD x rain + rain^2^ + temp | 933.11 | 0.17 | 0.53 |
| Base + rain + rain^2^ | 947.61 | 0.10 | 0.52 |
| Base + rain | 953.63 | 0.08 | 0.52 |
| Base | 959.99 | 0.05 | 0.52 |
| *Recruitment success* |  |  |  |
| Base + rain | 838.23 | 0.06 | 0.25 |
| Base | 844.11 | 0.04 | 0.25 |
| *Male parental survival* |  |  |  |
| Base + fledgling x rain | 834.36 | 0.05 | 0.05 |
| Base + rain | 838.60 | 0.03 | 0.03 |
| Base | 841.12 | 0.02 | 0.03 |

Barton K (2013) MuMIn: multi-model inference. r-package, version 1.10.5. http://cran.r-project.org/package=MuMIn

Nakagawa, S, Schielzeth, H. (2013). A general and simple method for obtaining R² from Generalized Linear Mixed-effects Models. *Methods in Ecology and Evolution*, 4, 133-142.
